# Retraction Note: Targeting 17q23 amplicon to overcome the resistance to anti-HER2 therapy in HER2+ breast cancer

**DOI:** 10.1038/s41467-026-74645-1

**Published:** 2026-06-29

**Authors:** Yunhua Liu, Jiangsheng Xu, Hyun Ho Choi, Cecil Han, Yuanzhang Fang, Yujing Li, Kevin Van der Jeught, Hanchen Xu, Lu Zhang, Michael Frieden, Lifei Wang, Haniyeh Eyvani, Yifan Sun, Gang Zhao, Yuntian Zhang, Sheng Liu, Jun Wan, Cheng Huang, Guang Ji, Xiongbin Lu, Xiaoming He, Xinna Zhang

**Affiliations:** 1 Department of Medical and Molecular Genetics, Indiana University School of Medicine, Indianapolis, IN 46202 USA; 2 Department of Cancer Biology, The University of Texas MD Anderson Cancer Center, Houston, TX 77030 USA; 3 Institute of Digestive Diseases, Longhua Hospital, Shanghai University of Traditional Chinese Medicine, 200032 Shanghai, China; 4 Department of Biomedical Engineering and Comprehensive Cancer Center, The Ohio State University, Columbus, OH 43210 USA; 5 Fischell Department of Bioengineering, University of Maryland, College Park, MD 20742 USA; 6 Department of Electronic Science and Technology, School of Information Science and Technology, University of Science and Technology of China, 230027 Hefei, China; 7 Drug Discovery Laboratory, School of Pharmacy, Shanghai University of Traditional Chinese Medicine, 201203 Shanghai, China; 8 Indiana University Melvin and Bren Simon Cancer Center, Indiana University School of Medicine, Indianapolis, IN 46202 USA; 9 Robert E. Fischell Institute for Biomedical Devices, University of Maryland, College Park, MD 20742 USA; 10 Marlene and Stewart Greenebaum Comprehensive Cancer Center, University of Maryland, Baltimore, MD 21201 USA; 11 The Center for RNA Interference and Non-coding RNAs, The University of Texas MD Anderson Cancer Center, Houston, 77030 TX USA; 12 Department of Gynecologic Oncology and Reproductive Medicine, The University of Texas MD Anderson Cancer Center, Houston, TX 77030 USA

Retraction to: *Nature Communications* 10.1038/s41467-018-07264-0, published online 09 November 2018

This paper is being retracted after a joint research misconduct investigation conducted by Indiana University, The Ohio State University, and the University of Maryland, College Park found that Figure 5a/b/d/e and Supplementary Fig. 9 contain irregularities. The Editor has therefore lost confidence in this paper.

Yuanzhang Fang did not explicitly state whether or not they agree with this retraction.

Xiongbin Lu, Xinna Zhang, Xiaoming He, Jiangsheng Xu disagree with this retraction.

Yunhua Liu, Hyun Ho Choi, Cecil Han, Yujing Li, Kevin Van der Jeught, Hanchen Xu, Lu Zhang, Michael Frieden, Lifei Wang, Haniyeh Eyvani, Yifan Sun, Gang Zhao, Yuntian Zhang, Sheng Liu, Jun Wan, Cheng Huang, and Guang Ji did not respond to correspondence from the Publisher about this retraction.

